# Plantago asiatica mosaic virus: An emerging plant virus causing necrosis in lilies and a new model RNA virus for molecular research

**DOI:** 10.1111/mpp.13243

**Published:** 2022-07-20

**Authors:** Ken Komatsu, John Hammond

**Affiliations:** ^1^ Graduate School of Agriculture Tokyo University of Agriculture and Technology (TUAT) Fuchu Japan; ^2^ US Department of Agriculture Agricultural Research Service (USDA‐ARS) Beltsville Maryland USA

**Keywords:** lily, *Plantago asiatica*, potexviruses, systemic necrosis, *Tymoviridae*

## Abstract

**Taxonomy:**

*Plantago asiatica mosaic virus* belongs to the genus *Potexvirus* in the family *Alphaflexiviridae* of the order *Tymovirales*.

**Virion and genome properties:**

Plantago asiatica mosaic virus (PlAMV) has flexuous virions of approximately 490–530 nm in length and 10–15 nm in width. The genome of PlAMV consists of a single‐stranded, positive‐sense RNA of approximately 6.13 kb. It contains five open reading frames (ORFs 1–5), encoding a putative viral polymerase (RdRp), movement proteins (triple gene block proteins, TGBp1‐3), and coat protein (CP), respectively.

**Host range:**

PlAMV has an exceptionally wide host range and has been isolated from various wild plants, including *Plantago asiatica*, *Nandina domestica*, *Rehmannia glutinosa*, and other weed plants. Experimentally PlAMV can infect many plant species including *Nicotiana benthamiana* and *Arabidopsis thaliana*. It also infects ornamental lilies and frequently causes severe necrotic symptoms. However, host range varies depending on isolates, which show significant biological diversity within the species.

**Genome diversity:**

PlAMV can be separated into five clades based on phylogenetic analyses; nucleotide identities are significantly low between isolates in the different clades.

**Transmission:**

PlAMV is not reported to be transmitted by biological vectors. Virions of PlAMV are quite stable and it can be transmitted efficiently by mechanical contact.

**Disease symptoms:**

PlAMV causes red‐rusted systemic necrosis in ornamental lilies, but it shows much weaker, if any, symptoms in wild plants such as *P. asiatica*.

**Control:**

Control of the disease caused by PlAMV is based mainly on rapid diagnosis and elimination of the infected bulbs or plants.

## Graphical abstract

This pathogen profile summarizes current knowledge regarding Plantago asiatica mosaic virus, including its host range, strain differentiation, host interactions, and utility as a viral vector to examine virus–host interactions.

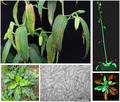

## 1 INTRODUCTION

Plantago asiatica mosaic virus (PlAMV), genus *Potexvirus*, is an emerging virus originally described in 1976 from the weedy plant *Plantago asiatica* in the Russian Far East (Kostin & Volkov, 1976) and the cultivated plant *Nandina domestica* in California (Moreno et al., 1976; Zettler et al., 1980). For almost three decades no other natural hosts were known until infections of edible lilies were reported in Japan and subsequently in commercially produced ornamental lilies in the Netherlands. Since then, PlAMV infection has become widespread through the commercial lily trade, and additional natural hosts have been reported from various countries.

Over the last two decades there has been significant work on molecular and biological characterization of PlAMV. Here we summarize current knowledge regarding PlAMV, including its natural and experimental host range, strain differentiation, host interactions, and utility as a plant viral vector to examine virus–host interactions.

## 2 HOST RANGE, TRANSMISSION, AND SYMPTOMS

### 2.1 Natural host range

PlAMV was first reported from *P. asiatica*, a perennial herbaceous species endemic to north‐eastern Asia, by Kostin and Volkov (1976). *P. asiatica* readily establishes in disturbed soils and can be a weed in fields and gardens. For the next 25 or more years, *P. asiatica* was the only known natural host. However, a potexvirus was reported to infect cultivated plants of the ornamental shrub *N. domestica* (heavenly bamboo) in California, USA (Moreno et al., 1976), and later was named Nandina mosaic virus (Zettler et al., 1980). Finally, it was classified as an isolate of PlAMV when its complete genome sequence was determined (Hughes et al., 2005).

Additional natural hosts began to emerge in the early 2000s. Reports of PlAMV infection in edible lilies (*Lilium leichtlinii* var. *maximowiczii*) in Japan (Komatsu et al., 2008; Ozeki et al., 2006; Sasaki, 2008) and *Primula sieboldii* (Komatsu et al., 2008) were followed later by the emergence of PlAMV in the commercial lily trade. This was reported first in the Netherlands (EPPO, 2011) and in Chile in 2013 (Vidal et al., 2016). However, soon after its first known occurrence in commercial lily hybrids, PlAMV was detected in commercial lily stocks in many other countries in Europe and around the world (Anderson et al., 2013; Chen et al., 2013; Hammond et al., 2015; Harju et al., 2018; Kim et al., 2015; Li et al., 2017; Montero‐Astúa et al., 2017; Pájtli et al., 2015; Parrella et al., 2015; Xu et al., 2017). Interestingly, the isolates from commercial lily products (“European‐like” isolates) have extremely closely related sequences, quite distinct from those obtained from edible lilies in Japan (Hammond & Reinsel, 2018; Komatsu et al., 2017; Ozeki et al., 2006).

Further isolates were found in other plant species and countries, including *P. asiatica* in Korea (Lim et al., 2016), *N. domestica* and *Viola grypoceras* in Japan (Komatsu et al., 2017), *Rehmannia glutinosa* in Korea (Kwak et al., 2018) and Japan (Uehara‐Ichiki et al., 2018), *Achyranthes bidentata* and *Stellaria* sp. in Japan (authors' unpublished data), and *Stellaria media*, *Primula vulgaris*, and *Urtica urens* in the Netherlands (De Kock, Kok, et al., 2013). Nucleotide sequences, but as yet no published reports, indicate infections of *Digitalis purpurea* (LC667833) and *Pelargonium inquinans* (LC667834) in Korea, and *Epimedium* sp. (MZ344590) in Canada. The natural hosts are shown in bold in Table 1.

**TABLE 1:** Host range of Plantago asiatica mosaic virus (PlAMV)

Family	Species	Common name	PlAMV local	PlAMV upper	Reference
**Plantaginaceae**	***Plantago asiatica***		+	+	Kostin and Volkov (1976)
	*Plantago lanceolata*	Ribwort plantain	+	(+)	Hammond and Rane (2022), Kostin and Volkov (1976)
	*Antirrhinum majus*	Snapdragon	+	−	Hammond and Rane (2022 )
**Berberidaceae**	***Nandina domestica***	Heavenly bamboo, nandina	+	+	Moreno et al. (1976)
**Liliaceae**	***Lilium leichtlinii* var. *maximowiczii***	Edible Asiatic lily	+	+	Ozeki et al. (2006)
	***Lilium* hybrids**	Ornamental lilies (Asiatic, Oriental, and tiger lilies)	+	+	Anonymous (2010), Hammond (2018)
**Primulaceae**	***Primula sieboldii***	Siebold primrose	+	+	Komatsu et al. (2008)
	*Primula acaulis*	Primrose	+	+	Hammond and Rane 2022()
**Urticaceae**	***Urtica urens***	Annual nettle	+	+	Hammond (2018)
**Orobanchaceae**	***Rehmannia glutinosa***	Chinese foxglove	+	+	Kwak et al. (2018)
**Caryophyllaceae**	***Stellaria media***	Common chickweed	+	+	Hammond (2018)
	*Dianthus superbus*	Large pink	+	+	Kostin and Volkov (1976)
**Violaceae**	***Viola grypoceras***	Cyclamen‐leaved violet	+	+	Komatsu et al. (2017)
	*Viola wittrockiana*	Pansy	+	+	Hammond and Rane (2022)
**Amaranthaceae**	***Achyranthes bidentate* var*. fauriei***	Ox knee	+	+	Hammond (2018)
	*Amaranthus albus*	Pigweed amaranth	+	−	Kostin and Volkov (1976)
	*Amaranthus paniculatus*	Red amaranth	+	−	Kostin and Volkov (1976)
	*Amaranthis retroflexus*	Redroot amaranth	+	+	Kostin and Volkov (1976)
	*Atriplex hortensis*	Garden orache	+	−	Kostin and Volkov (1976)
	*Celosia spicata*	Wheat celosia	+	+	Hammond and Rane (2022)
	*Gomphrena globosa*	Annual globe amaranth	+	−	Kostin and Volkov (1976)
	*Gomphrena haageana*	Perennial globe amaranth	+	+	Hammond and Rane (2022)
Solanaceae	*Nicotiana benthamiana*		+	+	Ozeki et al. (2006)
	*Nicotiana edwardsonii*		+	+	Hammond and Rane (2022)
	*Nicotiana. megalosiphon*		+	+	Hammond and Rane (2022)
	*Nicotiana occidentalis*		+	+	Hammond and Rane (2022)
	*Physalis alkekengi* var. *franchetii*	Chinese lantern	+	+	Hammond and Rane (2022)
	*Salpiglossis* (hybrid)	Stained glass flower	(+)	−	Hammond and Rane (2022)
	*Solanum lycopersicum*	Tomato	+	+	Hammond and Rane (2022)
Asteraceae	*Centaurea cyanus*	Bachelor's button	+	−	Hammond and Rane (2022)
	*Echinacea purpurea*	Purple coneflower	(−)	+	Hammond and Rane (2022)
	*Tagetes patula*	Mexican marigold	+	−	Hammond and Rane (2022)
	*Zinnia elegans*	Zinnia	+	−	Hammond and Rane (2022)
	*Emilia coccinea* (syn. *E. sagittata*)	Scarlet tasselflower	+	+	Kostin and Volkov (1976)
	*Xanthium strumarium* (syn. *X. sibiricum*)	Rough cocklebur	+	+	Kostin and Volkov (1976)
Lamiaceae	*Monarda hybrida*	Lambada bee balm	+	+	Hammond and Rane (2022)
	*Ocimum basilicum*	Basil	+	+	Kostin and Volkov (1976)
Polygonaceae	*Fagopyrum sagittatum*	Buckwheat	+	+	Kostin and Volkov (1976)
	*Persicaria orientalis* (syn. *Polygonum orientale*)	Kiss me over the garden gate	+	+	Kostin and Volkov (1976)
	*Polygonum* sp.		+	+	Kostin and Volkov (1976)
Polemoniaceae	*Phlox drummondii*	Annual phlox	+	+	Hammond and Rane (2022 )
Chenopodiaceae	*Chenopodium quinoa*	Quinoa	+	+	Komatsu et al. (2017)
	*Chenopodium album* (syn. *Chenopodium amaranticolor*)	Lambsquarters	+	−	Kostin and Volkov (1976)
	*Chenopodium glaucum*	Oak‐leaved goosefoot	+	−	Kostin and Volkov (1976)
	*Chenopodium* sp.		+	+	Kostin and Volkov (1976)
Tropaeolaceae	*Tropaeolum minus*	Nasturtium	+	−	Hammond and Rane (2022)
Campanulaceae	*Platycodon grandiflorus*	Balloon flower	+	+	Kostin and Volkov (1976
Balsamineaceae	*Impatiens walleriana*	Impatiens	+	+	Hammond and Rane (2022)
Cleomaceae	*Cleome hasslerana*	Cleome, spiderflower	+	−	Hammond and Rane (2022)
Brassicaceae	*Lobularia maritima*	Sweet allysum	+	−	Hammond and Rane (2022)
	*Arabidopsis thaliana*	Thale cress	+	+	Minato et al. (2014
Papaveraceae	*Papaver* sp.	Poppy	+	+	Kostin and Volkov (1976)
Fabaceae	*Vicia faba*	Broad bean	+	−	Kostin and Volkov (1976)
	*Trifolium incarnatum*	Crimson clover	+	+	Kostin et al. (1976)
Ranunculaceae	*Clematis terniflora* var. *mandshurica* (= *Clematis mandshurica*)	Sweet autumn clematis	+	+	Kostin and Volkov (1976)
Aizoaceae	*Tetragonia tetragonioides* (syn. *Tetragonia expansa*)	New Zealand spinach	+	+	Kostin and Volkov (1976)

*Note*: Host range is from experimental inoculation by Hammond and Rane (2022) unless another reference is noted; host species and plant families found naturally infected are shown in bold font.

### 2.2 Experimental host range

When PlAMV was first discovered in *P. asiatica*, a partial experimental host range was determined, including 24 species from 12 plant families (Kostin & Volkov, 1976; Table 1). However, it remained unknown whether other isolates have a similar host range or not. Recently, the experimental host range of a lily isolate, one of the “European‐type” PlAMV isolates in commercial lilies, was determined (Hammond & Rane, 2022), which identified an additional 20 species representing 12 taxonomically diverse plant families not previously reported (Kostin & Volkov, 1976; Zettler et al., 1980; see Table 1). Another recent study showed that PlAMV isolates in distinct phylogenetic clades show differential infectivity to several experimental hosts. Among five isolates tested, only two isolates each can systemically infect *Arabidopsis thaliana* or *P. asiatica* (authors' unpublished data). Several experimental hosts listed in Table 1 as either locally or systemically susceptible to a lily PlAMV isolate were reported as not susceptible to a nandina isolate (Zettler et al., 1980). Collectively, these findings suggest that PlAMV has a wide host range but that different isolates vary in their ability to infect some hosts.

### 2.3 Transmission

No biotic vector of PlAMV is known. As with other potexviruses, PlAMV is readily transmitted by mechanical inoculation with sap extracts (Conijn, 2014; De Kock, 2013), but also spreads rapidly between infected and previously healthy lilies planted in a common container by uptake and probably exudation through the roots, and is remarkably stable in contaminated planting media (Conijn, 2014; De Kock, 2013). PlAMV is also transmitted between lilies during bulb washing and packing, which may be the major route of infection in commercial lilies (Chastagner et al., 2017; De Kock, Kok, et al., 2013; De Kock, Slootweg, et al., 2013). Fields in which PlAMV‐infected lilies were previously grown can retain viable virus, able to infect up to 8% of lily stocks previously thought to be free from PlAMV (De Kock, Slootweg, et al., 2013). PlAMV can also systemically infect lily plants by mechanical inoculation or sap injection into stems (Tanaka et al., 2019).

### 2.4 Symptoms

**FIGURE 1:**
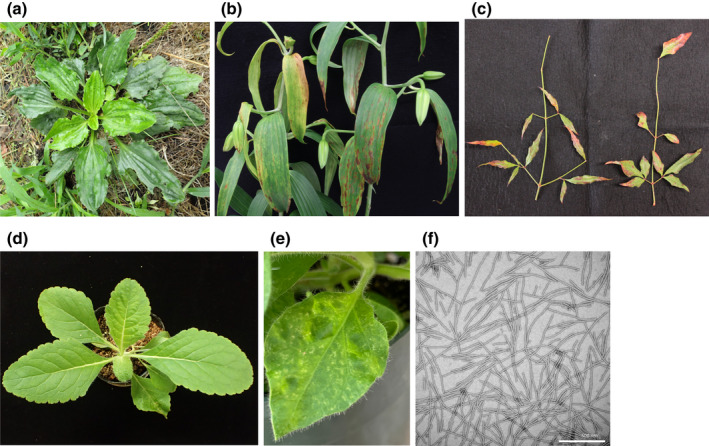
Symptoms associated with Plantago asiatica mosaic virus (PlAMV) infection. (a) *Lilium* spp. plants showing red‐coloured necrotic streaking in the whole plant. (b) *Plantago asiatica* plants showing mild mottling symptoms. (c) Leaves of *Nandina domestica* plants showing leaf narrowing. (d) *Rehmannia glutinosa* plants showing no clear symptoms. (e) A leaf of *Nicotiana edwardsonii* showing mottling and chlorotic spots. (f) Transmission electron microscope image of PlAMV virions. Scale bar 500 nm.

The natural hosts of PlAMV vary in the degree of symptom expression. *P. asiatica* symptoms are mottling, mottled chlorosis (e.g., Lim et al., 2016; authors' unpublished data; Figure 1a), or inconspicuous. Similarly, other PlAMV‐infected species may show minimal symptoms under some environmental or nutritional conditions. Symptoms on lily cultivars may vary widely, with some showing only mild foliar mottling while others show necrotic spotting or streaking on both foliage and sepals (Figure 1b), and in some cases also on the flowers. As lilies are commonly also infected with cucumber mosaic virus (CMV), lily symptomless virus (LSV), and/or lily mottle virus (LMoV), it is not clear whether the variability in symptoms is more dependent on cultivar, the environment, or interactions with other viruses. However, symptom severity is enhanced by significant temperature fluctuations (iBulb, 2016), and mixed infections with LSV and LMoV can result in much more severe symptoms, including significant stunting (Chastagner et al., 2017; Sasaki, 2008). Symptoms in PlAMV‐infected lilies may also be mistaken for severe nutrient deficiency or chemical phytotoxicity (Chastagner et al., 2017).

Small differences in the PlAMV genome can affect symptoms in model plants. Six distinct isolates (Li1–Li6) were obtained from a single infected *L. leichtlinii* var. *maximowiczii* plant following multiple single local lesion transfers in *Tetragonia expansa* (Komatsu et al., 2008). Isolates Li1 and Li6 differed significantly in symptom production in *Nicotiana benthamiana*, with Li1 inducing necrotic local lesions, leading to systemic necrosis; in contrast Li6 caused no symptoms in either inoculated or systemically infected leaves (Ozeki et al., 2006). Infectious clones of Li1 and Li6 had no appreciable differences in the speed of virus systemic movement; however, Li1 RNA accumulation exceeded that of Li6 (Ozeki et al., 2006). Exchange of a single amino acid residue in the replicase differing between Li1 and Li6 approximately equalized RNA concentrations of the mutants, but caused a reversal of symptom type, indicating that symptom type is not correlated with replication levels (Ozeki et al., 2006).

Two PlAMV cultures were isolated in the United States from different lily cultivars and maintained by serial passage in *N. benthamiana* without single lesion passaging. Each showed varying symptoms within and between single plants of *N. benthamiana*, typically chlorotic to necrotic local lesions, followed by systemic mottle or mosaic. Some leaves with mosaic developed necrotic patches, often spreading down the veins into the petiole and the main stem; other upper leaves developed narrow areas of white tissue surrounding areas of mosaic (authors' unpublished data). This suggests the presence of a mixture of sequence variants that compete for predominance within the same plant, similar to the occurrence of Li1–Li6 in *L. leichtlinii* or variants of Alternanthera mosaic virus (AltMV) in *Phlox stolonifera* (Lim et al., 2010).

Naturally infected *V. grypoceras* showed obvious mosaic symptoms (Komatsu et al., 2017). *N. domestica* from Japan showed primarily leaf narrowing (Figure 1c; Komatsu et al., 2017), while that found in the United States showed the systemic mosaic without leaf distortion on the first or second leaves produced after inoculation, and intermittently on subsequently developing nandina leaves (Zettler et al., 1980). PlAMV infecting *R. glutinosa* from both Korea and Japan was always found in combination with other viruses (Kwak et al., 2018; Uehara‐Ichiki et al., 2018). Because PlAMV inoculated to virus‐free *R. glutinosa* yielded no clear symptoms, mosaic, veinal necrosis, and chlorotic or necrotic local spots reported in naturally infected plants were caused by coinfection with another virus (Figure 1d; T. Uehara‐Ichiki, National Agriculture and Food Organization [NARO], Ibaraki, Japan, personal communication). Naturally infected *Achyranthes bidentata* and *Stellaria* sp. found in Japan showed only mild mosaic, barely distinguishable from asymptomatic plants (authors' unpublished data).

Some experimental hosts were systemically infected latently, whereas others were infected locally but not systemically, either with or without any obvious symptoms. Several *Nicotiana* species, notably *N. benthamiana*, *N. edwardsonii*, *N. megalosiphon*, and *N. occidentalis*, developed clear systemic mosaic that frequently became necrotic in a significant proportion of the leaves, with symptom severity varying both within and between individual plants (Figure 1e). *Plantago lanceolata* became systemically infected without obvious symptoms, but in contrast to other systemic hosts, including *Monarda hybrida* and *Celosia spicata*, the level of virus significantly declined over time (Hammond & Rane, 2022; authors' unpublished data).

## 3 TAXONOMY AND GENOME DIVERSITY OF ISOLATES

### 3.1 Taxonomy of the species

*Pantago asiatica mosaic virus* is a member of the genus *Potexvirus* in the family *Alphaflexiviridae*, in the order *Tymovirales*. The flexuous PlAMV virions are approximately 490–530 nm long and 10–15 nm wide (Figure 1f). Its genome contains five open reading frames (ORFs), characteristic of potexviruses. Phylogenetic analysis based on the amino acid sequence of its replicase revealed that PlAMV is related to other potexviruses, including tulip virus X (TVX), hosta virus X, and hydrangea ringspot virus. TVX, which also infects ornamental plants including tulips, lilies, and lemon balm, is the most closely related (Tzanetakis et al., 2005; Yamaji et al., 2001). The nucleotide identity of the whole genome between PlAMV and TVX is almost 70%, below the demarcation criteria for distinct potexvirus species (72%), but it is one of the highest identities between different species of the genus (Komatsu et al., 2008; Yamaji et al., 2001). TVX has recently been detected from ornamental lily cultivars from which PlAMV has been repeatedly detected (Jo & Cho, 2018). Although there are no reports of intermediate virus isolates of these closely related species, PlAMV and TVX may be considered as a phylogenetically related group of monocot‐infecting potexviruses.

### 3.2 Genome diversity of PlAMV isolates

Phylogenetic analysis showed that PlAMV isolates affecting ornamental lilies worldwide (“European” isolates) are highly genetically homogenous, suggesting a common origin of these isolates (Hammond & Reinsel, 2018). However, PlAMV isolates, in general, have genomic diversity within the species. As stated above, PlAMV has been isolated from a variety of weed plants, including *P. asiatica* (Komatsu et al., 2008, 2017; Kostin & Volkov, 1976; Lim et al., 2016; Solovyev et al., 1994), *N. domestica* (Hughes et al., 2005; Komatsu et al., 2017), and *R. glutinosa* (Kwak et al., 2018; Uehara‐Ichiki et al., 2018). These PlAMV isolates from plants other than ornamental lilies share less than 85% nucleotide identities with lily‐infecting European isolates (Hammond & Reinsel, 2018; Komatsu et al., 2017), therefore the ancestral host plant from which lily‐infecting isolates were derived is still unclear. Our phylogenetic analysis based on the full‐length genomic sequences of PlAMV isolates showed that they were divided into five distinct clades according to their geographical origins and host plants (authors' unpublished data). Nucleotide identities between PlAMV isolates belonging to different clades are less than 85%. Sequence variability is dispersed throughout the genome, while several insertions/deletions of amino acids were concentrated within the linker region between methyltransferase and helicase domains of the replicase (Komatsu et al., 2017). Recent study has also revealed several positively selected amino acid residues in PlAMV‐encoded proteins, including this linker region (authors' unpublished data). Further studies are needed to identify specific amino acids contributing to intraspecies diversification and adaptation to ornamental lilies, and to understand the evolutionary history of PlAMV leading to genetic diversification within the species.

## 4 GENOME ORGANIZATION AND PROTEINS

### 4.1 Genome organization and gene expression

**FIGURE 2:**
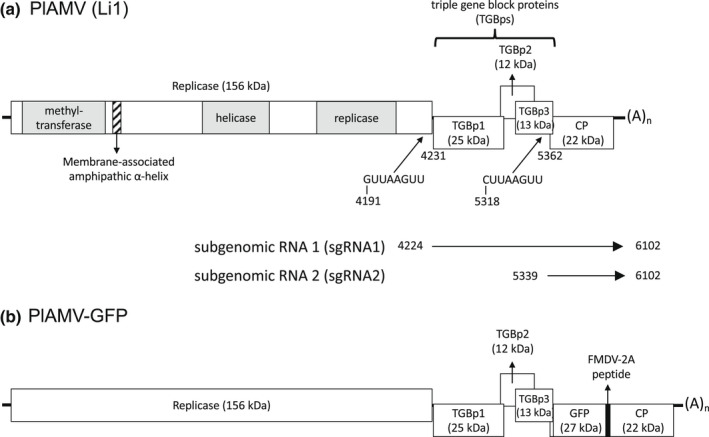
Schematic representation of genome organization and gene expression strategy of Plantago asiatica mosaic virus (PlAMV). (a) Genome organization and gene expression strategy of PlAMV (Li1 isolate: AB360790). Open reading frames (ORFs) are represented by open boxes. Untranslated regions are represented by straight lines. The protein encoded by each ORF and its predicted molecular weight (kDa) is shown inside or above the box. The sequence and positions of two putative promoter sequences for subgenomic RNAs (sgRNAs) are shown. Two sgRNAs produced during PlAMV infection are illustrated as horizontal arrows, with their transcription start sites shown previously (Fujimoto et al., 2022). Three conserved domains of the replicase are represented by grey boxes. A membrane‐associated amphipathic α‐helix located downstream from the methyltransferase domain is shown by a hatched box. (b) Genome organization of PlAMV vector expressing green fluorescent protein (GFP). A GFP cistron was translationally fused with the coat protein (CP) ORF through the foot‐and‐mouth disease virus (FMDV) 2A peptide.

Similar to other potexviruses, the genome of PlAMV has five ORFs (Figure 2a). The first ORF encodes a replicase required for virus replication, the second to fourth ORFs encode the triple‐gene‐block proteins (TGBps) required for cell‐to‐cell movement, and the last ORF encodes the coat protein (CP). A distinguishing feature of the PlAMV genome is the overlapping of ORF4 with ORF5, also found in other potexviruses including TVX. There are 5′‐ and 3′‐untranslated regions (UTRs) upstream of ORF1 and downstream of ORF5, respectively. The length and sequence of the 5′‐UTRs are well conserved within PlAMV isolates from different hosts, suggesting a critical role in the virus life cycle. There are several indels in the 3′‐UTRs among PlAMV isolates. As reported for the *Potexvirus* type species *Potato virus X* (PVX), stem‐loop structures were identified in both UTRs by RNA folding predictions, which may be important for replication. Indeed, a PlAMV “replicon”, which consists of only the replicase ORF flanked by 5′‐ and 3′‐UTRs, can produce minus‐strand genomic RNA (Komatsu et al., 2011). This indicates that both UTRs have essential *cis*‐elements required for interaction with the replicase, as is the case with PVX (Komarova et al., 2006; Kwon et al., 2005; Kwon & Kim, 2006; Park et al., 2013).

Mechanisms of gene expression of these five ORFs are basically similar to those reported in PVX (Verchot, 2021). Replicase is translated directly from genomic RNA (Yoshida et al., 2019), while other proteins are translated from three subgenomic RNAs (sgRNAs). However, recent study revealed that only two sgRNAs are detected from PlAMV‐infected plants, sgRNA1 (about 1.9 kb in length) and sgRNA2 (about 0.8 kb), and movement proteins TGBp1–TGBp3 are mainly translated from a single sgRNA1, which encodes TGBp1 as the 5′‐terminal ORF, by leaky scanning (Fujimoto et al., 2022; Figure 2a). This leaky scanning is promoted through a short 5′‐UTR of sgRNA1 and the Kozak sequence around its initiation codon (Fujimoto et al., 2022). Similar to other potexviruses, CP encoded in the most 3′‐terminal ORF5 is likely to be translated from sgRNA2.

Based on this genome organization and gene expression strategy, a green fluorescent protein (GFP)‐expressing vector of PlAMV, widely used as a model virus infecting *Arabidopsis*, was constructed by fusion of GFP with CP through foot‐and‐mouth disease virus 2A (FMDV‐2A) peptide (Minato et al., 2014). On infection with PlAMV‐GFP, GFP is expressed from sgRNA2 as a fusion protein (Figure 2b). An sgRNA duplication strategy, used for the development of other potexvirus vectors (Abrahamian et al., 2021), is difficult to apply to PlAMV due to its overlapping ORF4 and ORF5 (Minato et al., 2014).

### 4.2 Function of the encoded proteins

ORF1 encodes a replicase, which contains three conserved domains: a methyltransferase, a helicase, and an RNA‐dependent RNA polymerase (Figure 2a). Replicase has been shown to be the only protein involved in replication of PlAMV by agrobacterium‐mediated transient expression (agroinfiltration; Komatsu et al., 2011). As for other plant RNA viruses such as tomato mosaic virus, red clover necrotic mosaic virus, and tomato bushy stunt virus (Gursinsky et al., 2009; Iwakawa et al., 2007; Komoda et al., 2004), translation and replication of PlAMV was recapitulated by utilizing a cell‐free extract of evacuolated BY‐2 protoplasts (Yoshida et al., 2019). In this in vitro system, PlAMV replicase forms a high‐molecular‐weight complex, called the pre‐membrane‐targeting complex (PMTC), in soluble fractions. The PMTC is probably subsequently targeted to cellular membranes, possibly the endoplasmic reticulum (ER), to form a mature virus replication complex (VRC). Recently, membrane targeting was shown to be mediated by an amphipathic α‐helix located downstream from the methyltransferase domain (Figure 2a; Komatsu et al., 2021). GFP fusion to the methyltransferase domain forms a large perinuclear complex, possibly representing the VRC of PlAMV, which was disrupted by mutations in the conserved hydrophobic amino acids of this α‐helix. Interestingly, mutation of a proline residue of this membrane‐associated helix, which is strictly conserved in potexviruses and forms a kink in the helix, hinders virus replication but does not affect the formation of the large complex (Komatsu et al., 2021). This finding implicates the proline residue in the interaction of the amphipathic α‐helix with host factors required for the activation of the VRC. Replicase is also involved in the induction of programmed cell death (PCD) responses, as shown in Section 5.1.

ORF2, ORF3, and ORF4 encode TGBps required for cell‐to‐cell movement. Indeed, disruption of each of these TGBps by mutation of their initiation codon inhibits cell‐to‐cell movement of PlAMV (Ozeki et al., 2009; Yoshida et al., 2019). Moreover, as shown below in Section 5.2, TGBp1 functions as a viral suppressor of RNA silencing (VSR) by interfering with the amplification step of RNA silencing (Okano et al., 2014; Senshu et al., 2009). Although it remains unknown whether the VSR activity of TGBp1 is required for the cell‐to‐cell movement of PlAMV, as shown in PVX (Bayne et al., 2005), the stronger VSR activity of PlAMV compared with that of PVX may contribute to the greater stability of the PlAMV vector expressing a foreign gene (Minato et al., 2014). In contrast, mutation of the TGBp1 of AltMV significantly reduced VSR activity, making this virus a more efficient virus‐induced gene silencing vector (Lim et al., 2010).

ORF5 encodes the only structural protein, the CP. As with other potexviruses, the CP of PlAMV is involved in cell‐to‐cell movement (Ozeki et al., 2009). Mutational analyses combined with *trans*‐complementation have revealed that the N‐terminal 14 amino acids of PlAMV CP are dispensable for virion formation, but important for viral cell‐to‐cell movement. PlAMV CP interacts with TGBp1, but this interaction is not sufficient to confer cell‐to‐cell movement (Ozeki et al., 2009).

## 5 HOST DEFENCE RESPONSES AGAINST PlAMV

### 5.1 Systemic necrosis

In general, defence responses against plant viruses consist of those mediated by NLR (nucleotide‐binding and leucine‐rich repeat) proteins and RNA silencing (Moon & Park, 2016). To date, there have been no reports of an NLR gene that can completely inhibit infection of PlAMV. However, systemic necrosis, also called systemic hypersensitive response (SHR), caused by PlAMV has been well studied using *N. benthamiana* as a model plant.

Symptoms of PlAMV isolates Li1 and Li6 on *N. benthamiana* are strikingly different: Li1 causes systemic necrosis, while Li6 induces asymptomatic systemic infection (Ozeki et al., 2006). Li1‐induced necrosis does not prevent systemic infection of the virus, but exhibits defence‐related gene expression and PCD that were not observed in Li6‐infected plants (Komatsu et al., 2010). Gene knockdown analysis by tobacco rattle virus‐induced gene silencing revealed that systemic necrosis requires *NbSGT1*, *NbRAR1*, and *NbMAPKKKα*, a set of genes known to be involved in NLR‐mediated disease resistance against plant pathogens. *NbMAPKKKβ* and *NbMAPKKKγ* also function as positive regulators of PlAMV‐induced PCD (Hashimoto et al., 2012). These findings indicate that systemic necrosis is associated with defence responses against PlAMV, suggesting continuity between NLR‐mediated hypersensitive responses in incompatible plants and systemic necrosis in susceptible plants (Seo et al., 2006).

Inoculation of chimeric viruses between Li1 and Li6 showed that the systemic necrosis was determined by cysteine at amino acid residue 1154 of Li1 replicase (Ozeki et al., 2006). However, agroinfiltration studies revealed that the elicitor activity of PlAMV replicase resides in its helicase domain (HEL), not its RNA‐dependent RNA polymerase domain (POL) that contains the amino acid residue 1154 (Komatsu et al., 2011). Notably, the necrosis‐eliciting activity of HEL was also observed in Li6, and inducible‐expression analysis demonstrated that the necrosis was induced in a replicase dose‐dependent manner. The difference in symptoms between Li1 and Li6 may be attributed to the accumulation level of a non‐isolate‐specific elicitor HEL, with expression indirectly regulated by amino acid 1154 that controls replication (Komatsu et al., 2011).

The expression of necrotic symptoms induced by PlAMV may be affected by other viral‐encoded proteins because they can affect the accumulation level of replicase. Indeed, the PlAMV‐Li1 expressing GFP (Figure 2b) does not cause systemic necrosis in *N. benthamiana* (Minato et al., 2014). Reduced viral replication due to the GFP expression probably decreases the expression level of an elicitor HEL. Expression of necrotic symptoms can differ depending on environmental conditions, including temperature, as shown in inoculation tests to ornamental lilies (Tanaka et al., 2019).

### 5.2 RNA silencing

Plant viruses encode VSRs that inhibit various steps of host antiviral RNA silencing (Csorba et al., 2015). The first identified VSR of potexviruses was TGBp1 of PVX, which interferes with spread of the RNA silencing signal (Voinnet et al., 2000). TGBp1s of several potexviruses were shown to possess varying degrees of VSR activity, among which that of PlAMV isolate Li1 was relatively strong (Senshu et al., 2009). Functional analyses using TGBp1‐transgenic lines of *A. thaliana* plants and transient expression in *N. benthamiana* revealed that PlAMV TGBp1 interacts with RNA‐dependent RNA polymerase 6 (RDR6) and Suppressor of Gene Silencing 3 (SGS3), host antiviral factors required for the *trans*‐acting small interfering RNA synthesis pathway (Okano et al., 2014). The RDR6–SGS3 complex amplifies RNA silencing through generation of secondary small interfering RNAs and functions to repress infections of several plant viruses (Csorba et al., 2015; Yoshikawa et al., 2013). PlAMV probably counteracts host antiviral RNA silencing by suppressing the RDR6‐SGS3 amplification steps, although the exact roles of the RDR6‐SGS3 complex in PlAMV infection and its subcellular localization remain elusive. A previous study showed that TGBp1 of a nandina isolate of PlAMV localized to the nucleolus and that leucine residues at amino acids 86 and 89 are essential for nucleolar localization and efficiency of VSR activity (Lim et al., 2010). Further work is needed to reveal the relationship between subcellular localization of TGBp1 and its interaction with the RDR6‐SGS3 complex.

The molecular mechanisms underlying the stronger VSR activity of PlAMV TGBp1 compared with other potexviruses are poorly understood. Studies using PVX and its nonhost *A. thaliana* have demonstrated that DICER‐like proteins DCL2, DCL3, and DCL4, as well as ARGONAUTE proteins AGO2 and AGO5, restrict systemic infection of PVX (Brosseau & Moffett, 2015; Jaubert et al., 2011). Another study showed that susceptibility of *A. thaliana* to PVX varies depending on the natural variation of AGO2 (Brosseau et al., 2020). These findings suggest that TGBp1 of PlAMV, which can effectively infect *A. thaliana*, also inhibits activities of these DCLs and AGOs in addition to the RDR6–SGS3 complex.

In addition to a *dcl2/dcl4Arabidopsis* mutant that is more susceptible to multiple plant viruses, an *ago4* mutant was more susceptible to PlAMV infection, which suggests that DCL2/DCL4 and AGO4 restrict PlAMV infection (Brosseau et al., 2016). Functional analyses using transient expression assays demonstrated that cytosolic AGO4 is involved in this restriction (Brosseau et al., 2016). Experiments on the additional target(s) of PlAMV TGBp1, and comparison of VSR activity between PlAMV and PVX are needed to reveal the role of TGBp1 in successful viral infection.

## 6 RESISTANCE GENES EFFECTIVE AGAINST PlAMV

No cultivars of ornamental lilies, in which PlAMV causes severe economic losses, have yet been identified that are completely PlAMV‐resistant, although symptom expression and viral infectivity depend on the cultivars (Tanaka et al., 2019). In contrast, laboratory experiments that use PlAMV‐based GFP‐expression vector and the model plant species *A. thaliana* have identified several host genes that confer resistance against, or effectively suppress, PlAMV infection (Minato et al., 2014). These include dominant and recessive resistance genes as well as other defence‐related genes.

### 6.1 Dominant resistance genes

Jacalin‐type lectin required for potexvirus resistance 1 (JAX1) is a dominant resistance factor that restricts PlAMV at the single‐cell level (Yamaji et al., 2012). JAX1 is a noncanonical lectin‐type resistance protein, not a conventional NLR. An active JAX1 was found from five of 45 ecotypes of *A. thaliana*, including Bay‐0, by screening using PlAMV‐GFP. In PlAMV‐susceptible ecotype Col‐0, a premature termination codon in the first exon of the *JAX1* gene generates a truncated 36 amino acid protein instead of the full‐length 157 amino acid protein. A β‐glucuronidase (GUS)‐promoter assay showed *JAX1* to be highly expressed in the vascular tissue, completely inhibiting systemic infection of potexviruses. This vascular‐specific expression and complete inhibition of systemic viral infection resembles that of the jacalin‐type lectin gene *RTM1* of *A. thaliana*, which confers resistance against tobacco etch virus, but the spectrum of resistance of JAX1 and RTM1 was different; *JAX1* confers resistance against potexviruses in general, while *RTM1* is effective against potyviruses (Chisholm et al., 2001; Yamaji et al., 2012). An in vitro replication assay based on evacuolated BY‐2 protoplast extracts revealed that JAX1, but not RTM1, restricts replication of potexviruses by targeting the massive protein complexes required for viral replication (Yoshida et al., 2019). This targeting is mediated via interaction with the viral replicase, and a single amino acid substitution, Q336H, allows PVX infection in *JAX1*‐expressing plants (Sugawara et al., 2013). However, the same mutation in PlAMV severely decreases infectivity in plants either with or without JAX1, suggesting that JAX1‐mediated resistance does not easily produce resistance‐breaking viral variants (authors' unpublished data). Jacalin‐related lectin genes are widely found in plants and many are involved in disease resistance (Esch & Schaffrath, 2017). However, it remains to be determined whether the antiviral functions of jacalin‐related lectins, including JAX1 and RTM1, are conserved in other plants.

### 6.2 Recessive resistance genes

In addition to the dominant resistance genes, genetic screening of ethyl methyl sulfonate‐mutagenized *Arabidopsis* lines with PlAMV‐GFP revealed recessive resistance genes that encode a plant factor required for successful virus infection (Hashimoto, Neriya, Yamaji, et al., 2016). *EXA1* (essential for potexvirus accumulation 1) is the first identified recessive resistance gene against PlAMV infection and inhibits replication at the single‐cell level (Hashimoto, Neriya, Keima, et al., 2016). EXA1 contains a GYF domain and an eIF4E‐binding motif. As the translation initiation factor eIF4E is the best‐known recessive resistance gene against plant viruses, EXA1 may form a translation initiation complex with eIF4E and possibly exerts its function through regulation of translation of PlAMV replicase or of a host factor required for PlAMV replication (Hashimoto, Neriya, Keima, et al., 2016). *EXA1* orthologs are found in a wide range of plant species, including tomato, rice, and *N. benthamiana*, and knockdown of *EXA1* orthologs in tomato and *N. benthamiana* significantly reduced the accumulation of potexviruses and the related lolavirus. This restriction of viral infection is cancelled by complementation with the rice *EXA1* gene, indicating that the proviral function of EXA1 is conserved among a wide range of plants (Yusa et al., 2019). However, the effect of *EXA1* knockdown in *N. benthamiana* on virus accumulation differs depending on virus species, suggesting that EXA1 paralogs function redundantly in a virus‐specific manner. Interestingly, EXA1 (also referred to as PSIG1) was reported to restrict PCD during bacterial and oomycete infections (Matsui et al., 2017). Localization of PSIG1 (EXA1) to P‐bodies supports its role in the suppression of P‐body activity, such as translational arrest of viral genomic RNA or nonsense‐mediated decay (Mäkinen et al., 2017).

Another recessive resistance gene against PlAMV found from *A. thaliana* is *nCBP1*, an isoform of *eIF4E*, known to be the loss‐of‐susceptibility gene for multiple plant viruses (Hashimoto, Neriya, Yamaji, et al., 2016). nCBP1 is required for infection of plant viruses in the families *Alpha*‐ and *Betaflexiviridae*. Whereas nCBP1 is not required for replication at the single‐cell level, it is required for cell‐to‐cell movement of PlAMV (Keima et al., 2017). Accumulation of both TGBp2 and TGBp3 was decreased in the *ncbp* mutant, which causes the inhibition of cell‐to‐cell movement (Keima et al., 2017).

### 6.3 Other defence‐related genes

Similar to various other RNA viruses, potexviruses require intracellular membranes for replication. Confocal laser scanning microscopy has revealed that the replicase of PVX is associated with ER membranes (Tilsner et al., 2013). Similarly, membrane association is important for replication of PlAMV (Komatsu et al., 2021; Yoshida et al., 2019). Membrane‐associated replication can cause elevated membrane stress. Indeed, ER‐localized TGBp3 of PVX was shown to induce unfolded protein responses (UPR), enhancing protein‐folding capacity at ER, especially of the IRE1/bZIP60 pathway (Gaguancela et al., 2016). As well as the IRE1/bZIP60 pathway, the IRE1‐independent bZIP17 pathway functions to restrict early stages of PlAMV infection in *Arabidopsis* plants, indicating that the two arms of UPR signalling inhibit the accumulation of PlAMV (Gayral et al., 2020). Meanwhile, bZIP60 and bZIP28 induce genes that support PlAMV infection, suggesting that plants have intricate regulatory mechanisms of UPR on virus infection (Herath et al., 2020). Although the mechanisms of the viral restriction conferred by UPR signalling remain elusive, UPR may ensure the induction of defence‐related proteins by increasing the protein‐folding capacity of ER damaged by viral replication.

Another defence‐related gene involved in restricting PlAMV infection is *non‐expressor of PR proteins 1* (*NPR1*), a key regulator of defence gene expression in the salicylic acid pathway. A plant immune activator, acibenzolar S‐methyl (ASM), restricted PlAMV infection at a single‐cell level, which requires *NPR1* (Matsuo et al., 2019). In ASM‐mediated restriction of PlAMV infection, cell death was not induced and DICER‐like proteins DCL2, DCL3, and DCL4, critical factors of RNA silencing, were not required (Matsuo et al., 2019).

## 7 DETECTION AND CONTROL STRATEGIES

### 7.1 Detection

Specific detection of PlAMV infection is necessary because PlAMV may infect various hosts asymptomatically (Chastagner et al., 2017) or symptoms may be modified in mixed infections with other viruses (Chastagner et al., 2017; Kim et al., 2019; Kwak et al., 2018; Kwon et al., 2019; Sugiyama et al., 2008; Uehara‐Ichiki et al., 2018). However, although bioassays using suitable hosts, including *N. benthamiana*, *N. edwardsonii*, *Chenopodium quinoa*, *Chenopodium amaranticolor*, *Tetragonia tetragonioides*, and *Gomphrena globosa*, are helpful, they are time‐consuming (Hammond et al., 2015; Ozeki et al., 2006; Zettler et al., 1980). Instead, several specific reagents and methods have been developed for PlAMV detection based on serological and nucleic acid‐based techniques.

PlAMV‐specific polyclonal antisera have been prepared against the purified virus of either lily or nandina isolates (see Hammond, 2018) or against the bacterially expressed CP of a lily isolate (Chen et al., 2013). These antisera have been used for immunodiffusion tests (Zettler et al., 1980), direct tissue blotting, and indirect, antigen‐coated plate enzyme‐linked immunosorbent assay (ELISA; Chen et al., 2013), double‐antibody sandwich ELISA (DAS‐ELISA; e.g. Hammond et al., 2015; Parrella et al., 2015), or rapid lateral flow assays (LFAs). Some commercial agricultural diagnostic companies produce ELISA reagent kits and/or LFAs for PlAMV detection. For greatest sensitivity in DAS‐ELISA testing of lilies, testing leaves at the time of flowering, using leaves from about three‐quarters of the height of the flowering stem is recommended, although it can also be used on roots and bulb‐scales of stored bulbs preplanting. Notably, both ELISA and LFAs have been found to detect a wide variety of PlAMV isolates from different phylogenetic clades.

Multiple groups have reported reverse transcription‐polymerase chain reaction (RT‐PCR) detection of PlAMV, using either generic potexvirus primers (van der Vlugt & Berendsen, 2002) followed by sequencing or various PlAMV‐specific primers, mainly derived from the replicase‐ or the CP‐encoding regions (e.g., Chen et al., 2013; Hammond et al., 2015). RT‐PCR can detect PlAMV in some samples not detected by DAS‐ELISA (Hammond et al., 2015). Kim et al. (2019) incorporated a pair of PlAMV‐specific primers with primer sets specific for CMV, LMoV, and LSV to detect these four lily‐infecting viruses. Multiplex RT‐PCR assays have been developed to detect PlAMV, CMV, LMoV, and LSV in lilies (Xu et al., 2021), and PlAMV and four other viruses in *R. glutinosa* (Kwon et al., 2019). An immunocapture (IC)‐RT‐PCR assay, applied to detect three lily‐infecting viruses, CMV, LMoV, and LSV, is also promising (Zhang et al., 2017), but IC‐RT‐PCR has not been reported for PlAMV detection.

Real‐time quantitative RT‐PCR (RT‐qPCR) has also been used and is more suitable for quantifying PlAMV titre than other assays. Tanaka et al. (2019) developed a SYBR Green‐based RT‐qPCR assay based on primers from a conserved RdRp region and found that an isolate from Oriental lily (PlAMV‐OL) can infect ornamental lilies more efficiently than edible lily isolate Li1 (Tanaka et al., 2019). Furthermore, a multiplex TaqMan RT‐qPCR system for simultaneous detection of PlAMV, CMV, LSV, LMoV, and shallot yellow stripe virus in lilies has recently been reported (Xu et al., 2021). In this case, primers and probes were designed from conserved regions of the CP genes of each virus, and the probes for each virus were labelled with a different fluorescent dye. The sensitivity of the multiplex reaction was equal to that of each uniplex assay and can be applied for the comprehensive detection of viruses from lily production fields (Xu et al., 2021).

A reverse transcription loop‐mediated isothermal amplification (RT‐LAMP) assay was developed (Komatsu et al., 2015) and shown to detect diverse isolates of PlAMV with a 10‐fold increase in sensitivity over conventional RT‐PCR, without requiring RNA purification. Pricking the leaf sample with a toothpick, followed by dipping it into the reaction mix, resulted in reliable detection in field samples (Komatsu et al., 2015).

One of the most cost‐effective assays for simultaneous detection of PlAMV and other lily‐infecting viruses is a macroarray prepared on a nylon filter membrane with probes for each virus (PlAMV, CMV, LMoV, and LSV; Sugiyama et al., 2008), which showed similar or greater sensitivity than ELISA and correctly identified mixed infections.

High‐throughput sequencing has been used to identify PlAMV and any associated viruses infecting *P. asiatica* (Lim et al., 2016), lilies (e.g., Jo & Cho, 2018; Xu et al., 2017), and *R. glutinosa* (Uehara‐Ichiki et al., 2018), yielding several nearly complete genomes.

### 7.2 Control strategies

Control strategies against PlAMV are largely limited to the generation and selection of plant stocks free of PlAMV infection and avoidance of introduction of PlAMV. As PlAMV has no known biological vector (other than human trade in infected plant materials), pesticide applications are unlikely to control its spread.

Many countries have imposed strict standards for testing lily bulbs for import or export. Meristem tip culture, especially when combined with thermotherapy and/or chemotherapy, can result in recovering virus‐free plants. However, the plant material in tissue culture for international distribution or micropropagation should also be subjected to rigorous testing and selection. It is known that tissue culture itself can sometimes result in reduction of virus titre below the sensitivity of normal RT‐PCR detection. An initially undetectable virus titre can slowly build up over a number of weeks after acclimation of tissue‐cultured material to the greenhouse, therefore retesting plants after several weeks in the greenhouse is needed to select founder material for a nuclear stock.

PlAMV infection can be transferred to previously healthy lily bulbs during the washing and processing that occurs after bulb harvest (De Kock, Slootweg, et al., 2013). To minimize this possibility the bulb lots of highest quality should be treated before bulb lots known to have a higher prevalence of PlAMV infection; the processing equipment itself should also be decontaminated and the wash water treated to minimize transmission in washing subsequent lots (Chastagner et al., 2017; Conijn, 2014; De Kock, 2013; De Kock, Slootweg, et al., 2013). Frequent decontamination of the tools and equipment used at other stages of production is also recommended.

As PlAMV is highly stable and can also be retained in the soil or plant parts, planting in soil or growing medium in which infected plants have previously been grown should be avoided (Chastagner et al., 2017; De Kock, Slootweg, et al., 2013). Heating of contaminated planting medium for a sufficient time at a high temperature will inactivate PlAMV, with a temperature of 65°C maintained for 10 min recommended for bulb wash water (Conijn, 2014), if that is practical.

Weed control in fields where PlAMV‐infected plants are, or have been, grown is also important as a number of weed species have been found to maintain infectivity, as have volunteer plants regrowing after harvest of the crop (Chastagner et al., 2017; De Kock, Slootweg, et al., 2013). Maintaining fields fallow for one planting season may minimize sources of infection for the next crop (De Kock, Slootweg, et al., 2013). Moreover, the milled sphagnum used to pack lily bulbs for shipping has been proven to carry PlAMV (authors' unpublished data) and should be disposed of with caution to avoid contamination.

The possibility of using plant defence activators to minimize PlAMV infections has been studied by Matsuo et al. (2019) using ASM, a functional analog of salicylic acid, which can inhibit infection of tobacco mosaic virus (Chivasa et al., 1997; Murphy & Carr, 2002). Treating *N. benthamiana* with ASM prior to inoculation with PlAMV reduced the number of infection foci compared to controls, reflecting inhibition of replication, but did not affect cell‐to‐cell movement; however, there was a delay in long‐distance movement into the uninoculated leaves (Matsuo et al., 2019). Future work to further understand the mechanisms may lead to more effective prevention or minimization of the effects of plant virus infection.

## 8 CONCLUSION

The economic losses suffered in the ornamental lily industry and the rapid spread of PlAMV through the international trade in lily bulbs spurred interest in research into this rapidly emerging virus. To date, however, the natural host of origin of the strain in commercial lilies remains unidentified but seems to be derived from a single introduction.

The extent of the work on PlAMV that has resulted from this interest has revealed several features that make PlAMV an attractive model system to complement knowledge obtained from other well‐studied viruses in the genus *Potexvirus*. First, PlAMV has a diverse natural host range, encompassing both monocotyledonous and dicotyledonous species, whereas other “model” potexviruses infect either primarily monocots of the Poaceae (bamboo mosaic virus and foxtail mosaic virus) or dicots (AltMV, papaya mosaic virus, pepino mosaic virus, and PVX). Second, PlAMV shows multiple virus–host interactions involving various virus‐encoded proteins and five clades of diverse isolates spanning an array of natural host species. These should allow PlAMV gene exchanges to determine the factors affecting host range and symptom severity. Finally, a GFP‐labelled infectious clone was successfully developed to examine both similarities and differences of host interactions affecting levels of viral replication, cell‐to‐cell movement, and long‐distance movement in the same plant host.

The features of PlAMV summarized in this review therefore recommend PlAMV as a highly flexible model virus system, with an established knowledge base, suitable for addressing many questions in a wide variety of host plants and permitting commonalities and differences between monocot and dicot host systems to be probed with a single model virus.

## CONFLICT OF INTEREST

The authors declare no conflict of interest.

## Data Availability

Data sharing are not applicable to this article as no new data were created or analysed.
